# A Case of Transudative Chylothorax: A Diagnostic Dilemma

**DOI:** 10.7759/cureus.2247

**Published:** 2018-02-28

**Authors:** Kartikeya Rajdev, Akshay Avula, Dikshya Sharma, Wissam Mansour, Shivika Agarwal, Abdul Hasan Siddiqui, Michel Chalhoub

**Affiliations:** 1 Internal Medicine, Staten Island University Hospital, Northwell Health; 2 Pulmonary and Critical Care Medicine, Staten Island University Hospital, Northwell Health; 3 Medicine, Staten Island University Hospital, Northwell Health; 4 Internal Medicine, Faridabad, India; 5 Pulmonary and Critical Care Medicine, Staten Island University Hospital

**Keywords:** chylothorax, transudative, pleural effusion, idiopathic

## Abstract

Chylothorax is a type of pleural effusion characterized by the presence of chyle in the pleural space with triglyceride levels >110 mg/dL or evidence of chylomicrons in pleural fluid. Chylous effusion is typically exudative in nature with lymphocytic predominance. Transudative chylothorax is a rare finding which has been associated with only a limited range of clinical settings. We report a case of idiopathic transudative chylothorax for which the etiological cause could not be identified despite extensive workup and it resolved spontaneously after thoracentesis.

## Introduction

Chylothorax is defined as the accumulation of chyle in pleural space with a triglyceride level of more than 110 mg/dl or evidence of chylomicrons in the pleural fluid [1]. Chylothorax could be classified as either traumatic (thoracic surgery, central line placement, seat-belt trauma) or non-traumatic (malignancies usually lymphomas, metastatic cancer, superior vena cava (SVC) syndrome, venous thrombosis, and sarcoidosis) [2,3]. Typically chylous effusion is exudative in nature with lymphocytic predominance [4]. Transudative chylothorax is an extremely rare entity [5]. Based on the scarce literature available, transudative chylothorax has been ascribed to liver cirrhosis, nephrotic syndrome, amyloidosis, SVC thrombosis, congestive heart failure, and constrictive pericarditis [6]. We present a case of transudative chylothorax for which the etiological cause could not be established despite an extensive workup.

## Case presentation

**Figure 1:**
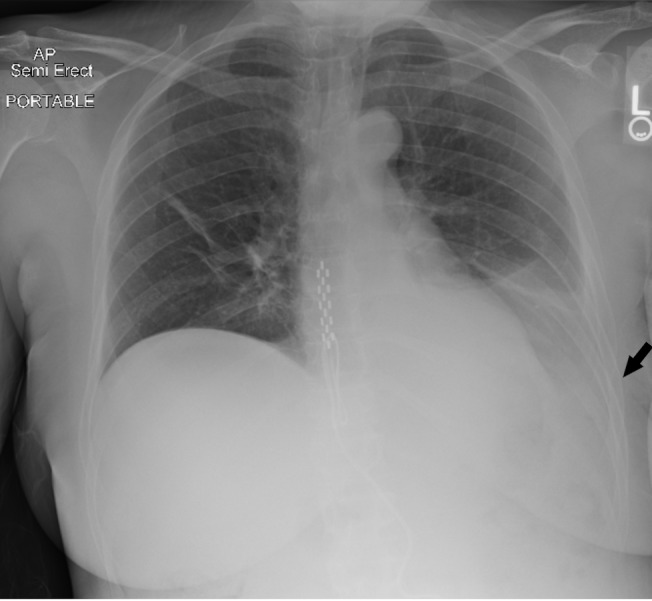
Chest X-ray on postoperative day 3 showing left-sided pleural effusion.

**Figure 2:**
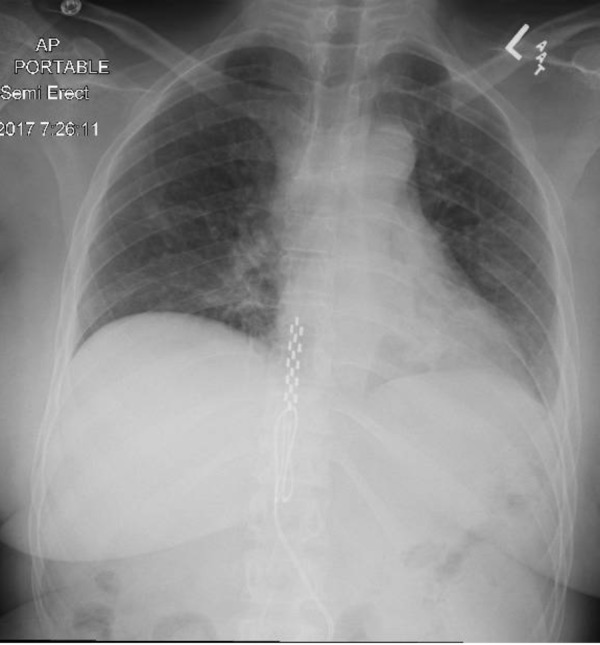
Chest X-ray before the surgery showing no pleural effusion.

**Figure 3:**
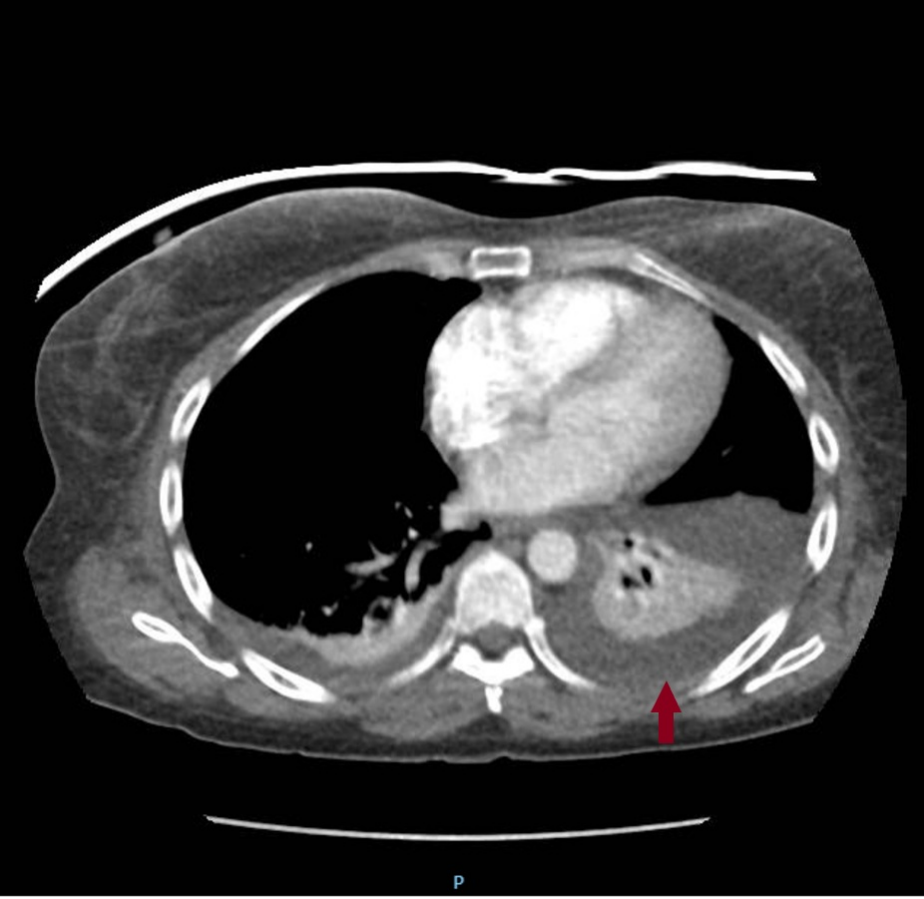
CT scan of chest showing left-sided pleural effusion. CT: Computed tomography

**Figure 4:**
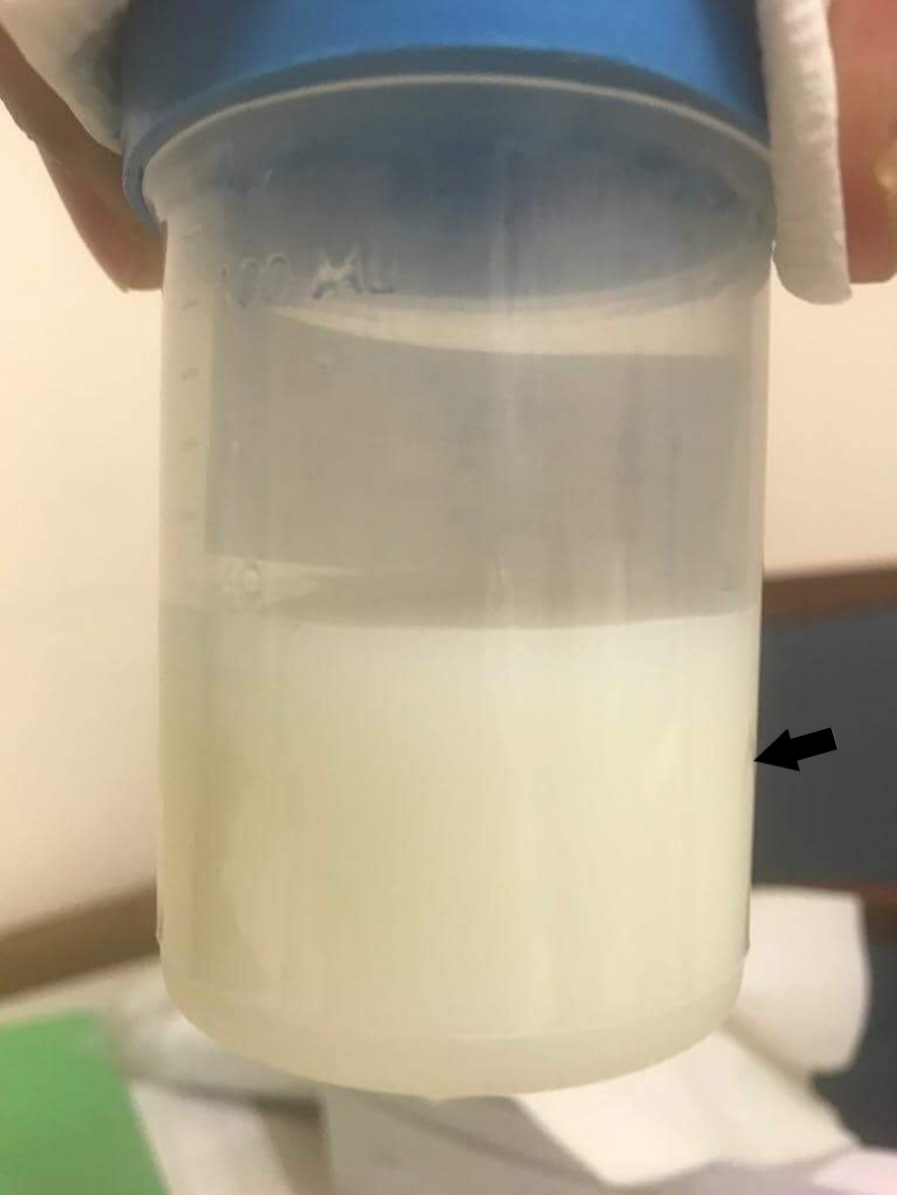
Turbid milky white pleural fluid immediately after thoracentesis.

A 53-year-old female, ex-smoker, was admitted to our hospital for an elective right sacroiliac (SI) joint fusion surgery for chronic pain and radiculopathy. Her past medical history was notable for asthma, depression, anxiety, fibromyalgia, bilateral sacroiliac joint fusion surgeries and placement of thoracic spinal cord stimulator for chronic pain. The patient’s SI joint fusion surgery was uneventful. On post-operative day 3, she dropped her oxygen saturation to 90% at rest and was found to have a new left side pleural effusion on the chest X-ray (CXR) (Figure 1). Her CXR before the surgery did not show pleural effusion (Figure 2). The patient was afebrile, denied any cough, chills or shortness of breath and there were no leukocytosis or any new laboratory abnormality. The examination was within normal limits except for decreased breath sounds on the left side of chest and did not reveal any cervical, axillary, or inguinal lymphadenopathy. Computed tomography (CT) scan of chest with intravenous contrast revealed moderate left-sided pleural effusion with compressive atelectasis along with small right-sided pleural effusion (Figure 3). No pulmonary embolism was detected on the CT scan. An ultrasound-guided thoracentesis was performed on the left side with drainage of milky white colored pleural fluid (Figure 4). Pleural fluid analysis revealed a lactate dehydrogenase (LDH) of 61 U/l and protein of <1 g/dl. Fluid triglyceride level was 385 mg/dl. Serum LDH was 236 U/l, serum triglyceride was 160 mg/dl, and serum protein was 3.6 g/dl. The fluid was transudative in nature according to lights criteria. Pleural fluid white blood cell (WBC) count was 229/microliter with neutrophil predominance (53%) followed by 45% macrophages and only 1% lymphocytes. Pleural fluid gram stain, bacterial culture, acid-fast bacterial stain and mycobacterial culture, adenosine deaminase level, and amylase were negative. Flow cytometry on the pleural fluid was negative for any lymphoma. An echocardiogram was within normal limits with an ejection fraction of 55-65%. Pulmonary artery pressures were within normal range with no signs of right ventricle dysfunction. Liver function test, ultrasound and CT scan of abdomen were within normal limits and did not show cirrhosis, ascites, pancreatitis, intra-abdominal mass or lymphadenopathy. Renal function and urine analysis were within normal limits. Serum antinuclear antibodies (ANA), antimitochondrial antibodies (AMA), antismooth muscle antibodies (ASMA), rheumatoid factor (RF), serum and urine protein electrophoresis, and hepatitis panel were also normal. Prior to discharge, a small pleural effusion was noted on the left side on the chest X-ray. A repeat CXR performed two weeks later showed complete resolution of the pleural effusion.

## Discussion

Chylothorax results from accumulation of chyle in the pleural space caused by disruption or obstruction in the flow of lymph along the thoracic duct [7]. Thoracic duct begins in the right side of abdomen at the level of second lumbar vertebrae and ascends through the diaphragm into the posterior mediastinum. At the level of fifth thoracic vertebrae, inclines towards the left side to enter the superior mediastinum and ends by draining into venous circulation at the angle of junction of the left subclavian vein with the internal jugular vein [7,8].

Typically, chylothorax is a type of exudative pleural effusion which is rich in triglycerides or shows the presence of chylomicrons. Chylothorax is usually characterized by all three of the following: 1) a triglyceride level of more than 110 mg/dL; 2) a ratio of pleural fluid to the serum triglyceride level of more than 1.0; and 3) a ratio of the pleural fluid to serum cholesterol level of less than 1.0 [1]. The presence of chylomicrons and triglycerides gives the turbid and milky appearance to the pleural fluid, however, chylothorax could also appear serous, serosanguinous or even bloody [9]. In a small minority of patients, the effusion could be transudative in nature. Transudative chylothorax is associated with cirrhosis, congestive heart failure, superior vena cava obstruction, nephrotic syndrome, and amyloidosis; however, it has been most commonly reported due to chyle leak associated with underlying liver cirrhosis [5,10]. The mechanisms for the development of transudative chylothorax have not been fully outlined. The traditional belief that chylothorax develops as a consequence of disruption/obstruction of lymphatic drainage along the thoracic duct does not entirely explain the events in case of transudative chylothorax. The suggested pathophysiology in transudative chylothorax includes translocation or leakage of chylous fluid or lymph across the diaphragm due to increased intra-abdominal pressures and in concurrence with degenerative changes in splanchnic lymphatics, seen in patients with cirrhosis or nephrotic syndrome [10]. It is also postulated that increase in pressure in pulmonary circulation and right side of heart leads to elevated back pressure into the thoracic duct and development of chylothorax. Reduction in the pressure in the system was noted to resolve the chylothorax [6,9]. Transudative chylothorax has been linked to only a sparse number of clinical settings as described above and recognition of these conditions in a timely manner can avoid expensive, unnecessary, and sometimes invasive workup in these patients.

In our case, extensive workup to identify the etiology of transudative chylothorax was inconclusive. It was interesting to note that the pleural fluid cell count in our patient was neutrophil predominant. The composition of chyle is normally lymphocytic predominant, low lactate dehydrogenase, low cholesterol and high protein level [4,7]. Neutrophil predominance is suggestive of the acute pleural process like infection or inflammation seen in parapneumonic effusion, empyema, pulmonary embolism, pancreatitis, etc. A rare case of neutrophilic transudative chylothorax has been reported in association with sclerosing mesenteritis by Rice et al. [7]. Our patient showed no evidence to suggest any underlying inflammatory or infectious process causing pleural effusion. Sacroiliac joint fusion surgery is unlikely to cause any disruption/obstruction of the thoracic lymphatics or increase in the pressure in thoracic duct. The etiology in our case remains a dilemma despite the temporal relation to the surgery.

## Conclusions

In summary, this report describes a case of a transudative chylothorax which is a rare finding. In this case, the etiological cause remained poorly understood and could not be elucidated other than the attribute that it developed post-operatively after an elective sacroiliac joint fusion surgery.
